# Circulation of *Dirofilaria immitis* and *Dirofilaria repens* in the Danube Delta Biosphere Reserve, Romania

**DOI:** 10.1186/s13071-018-2980-8

**Published:** 2018-07-04

**Authors:** Alexandru Tomazatos, Daniel Cadar, Edina Török, Iulia Maranda, Cintia Horváth, Lujza Keresztes, Marina Spinu, Stephanie Jansen, Hanna Jöst, Jonas Schmidt-Chanasit, Egbert Tannich, Renke Lühken

**Affiliations:** 1 Bernhard Nocht Institute for Tropical Medicine, WHO Collaborating Centre for Arbovirus and Hemorrhagic Fever Reference and Research, Hamburg, Germany; 2 Department of Biology and Ecology, Babeş-Bolyai University, Cluj-Napoca, Romania; 3 Romanian Academy Institute of Biology, Bucharest, Romania; 4 Molecular Biology Center, Institute for Interdisciplinary Research in Bio-Nano-Sciences, Babes-Bolyai University, Cluj-Napoca, Romania; 5 University of Agricultural Sciences and Veterinary Medicine, Cluj-Napoca, Romania; 6 Centre for Infection Research (DZIF), partner site Hamburg-Lübeck-Borstel-Riems, Hamburg, Germany

**Keywords:** *Dirofilaria repens*, *Dirofilaria immitis*, Prevalence, Vectors, Dogs

## Abstract

**Background:**

Dirofilariosis is an emerging vector-borne parasitic disease in Europe. Monitoring of wild and domestic carnivores demonstrated circulation of *Dirofilaria* spp. in Romania in the past. For the implementation of control measures, knowledge on the native mosquito community responsible for *Dirofilaria* spp. transmission is required.

**Methods:**

Mosquito samples originated from a longitudinal study previously performed in the Danube Delta Biosphere Reserve. Mosquito pools were screened for *Dirofilaria immitis* and *Dirofilaria repens*. The samples comprised 240,572 female mosquito specimens collected every ten days between April and September in 2014 at four different trapping sites. In addition, blood samples of 36 randomly selected dogs were collected in 2016 in each of the four mosquito sampling sites. A duplex real-time assay was used to detect the presence of one or both *Dirofilaria* species for each sample. This assay targets the cytochrome *c* oxidase subunit 1 and the *16S* rRNA gene fragments to differentiate both parasites.

**Results:**

*Dirofilaria immitis* and *D. repens* were detected in mosquito pools at all four trapping sites. In the 2118 mosquito pools tested, *D. immitis* was identified for eight and *D. repens* for six of the 14 screened mosquito taxa, with a higher prevalence of *D. immitis* (4.53% of analysed pools) compared to *D. repens* (1.09%). *Dirofilaria* spp. were also identified in dogs from the same sampling sites with a prevalence of 30.56%. For both *Dirofilaria* species, the highest estimated infection rates (EIRs) were found in *Anopheles maculipennis* (*s.l.*) (*D. immitis*: EIR = 0.206 per 100 specimens, *D. repens*: EIR = 0.066 per 100 specimens). In contrast, *Coquillettidia richiardii* and *Anopheles hyrcanus* as the most frequent taxa had infection rates which were significantly lower: *Cq. richiardii* (*D. immitis*: EIR = 0.021; *D. repens*: EIR = 0.004); *An. hyrcanus* (*D. immitis*: EIR = 0.028; *D. repens*: EIR = 0.006). The number of positive pools per calendar week was positively correlated with the number of screened pools per calendar week, suggesting constant *Dirofilaria* spp. transmission during the observation period.

**Conclusions:**

This study further confirms significant circulation of *Dirofilaria* spp. in eastern Europe, with high parasite prevalence in domestic canids and mosquitoes. Therefore, systematic monitoring studies are required to better understand the environmental risk factors for *Dirofilaria* transmission, allowing the implementation of effective surveillance and control measures.

## Background

*Dirofilaria repens* and *Dirofilaria immitis* are the causal agents of dirofilariosis in Europe [1]. Both vector-borne filarioid species (Nematoda: Onchocercidae) share the same transmission cycle, consisting of different mosquito species as intermediate hosts and canids as the predominant definitive hosts. The infection of pulmonary arteries and right heart chambers by *D. immitis* can cause severe conditions in dogs (heartworm disease) [2]. In contrast, *D. repens* in dogs are mainly detected in subcutaneous tissues with considerably milder symptoms (subcutaneous dirofilariosis). Other mammalian species, including humans, are aberrant hosts, in which the parasites’ fitness is reduced, resulting in the production of no or significantly fewer microfilariae [3]. Nevertheless, *Dirofilaria* spp. infections in humans can result in different symptoms. This predominantly includes local swelling caused by migration of the worm in the subcutaneous skin. However, in rare cases, there have been even reports of severe clinical manifestations including meningoencephalitis [4].

Human and canine dirofilariosis are considered emerging vector-borne parasitic diseases in Europe [2]. For decades, infections were predominantly restricted to Mediterranean regions and the eastern bounds of the continent. However, recent reports indicated a noticeable spread of the disease towards Central Europe. At the same time, an increasing numbers of human cases were reported for countries previously known for *Dirofilaria* spp. circulation (e.g. Ukraine [5], Bulgaria [6] and Belarus [7]).

*Dirofilaria* spp. were recognized in Romania at least a decade ago [8]. Different studies on the parasites’ prevalence in dogs detected local infection rates between 3% and even more than 60% [8–13]. In addition to domestic animals, recent studies also identified *D. immitis* or *D. repens* in different wild carnivore species (golden jackals, red fox, wildcat, grey wolf, least weasel), which probably have critical roles as reservoirs maintaining the helminths in natural disease foci [14, 15]. Nevertheless, only a few human cases have been identified in the country so far [16, 17]. A similar observation, i.e. high frequency of *Dirofilaria* spp. infections in carnivores in combination with low prevalence in humans, was also observed for other countries in eastern Europe (e.g. Belarus), which might be explained by a relative low awareness of physicians [7].

Nevertheless, although studies on vertebrate hosts have given clear indications for the autochthonous transmission of *Dirofilaria* spp. in Romania, there is a lack of knowledge regarding the native mosquito vectors. To the best of the authors’ knowledge, only one xenomonitoring study has been conducted so far. Ionică et al. [18] collected a relative small number of mosquitoes (~6000 specimens) in the Danube Delta Biosphere Reserve (DDBR). All samples of randomly collected mosquitoes were negative for filarioid DNA. Only a single *Aedes vexans* specimen was positive for *D. repens* trapped near a microfilaremic dog. The animal was co-infected with *D. immitis* and *D. repens*. However, as mentioned by the authors, the study results are not representative, as only a small number of specimens were collected on four days in July 2015.

Therefore, in order to get a comprehensive overview on the potential vectors of *Dirofilaria* spp., mosquito samples from a previous published longitudinal study in the DDBR [19] were screened for *D. immitis* and *D. repens* DNA. Lakes and channels in the area form a diverse mosaic of natural marshes. These provide highly diverse and productive breeding sites for mosquitoes. The samples from four trapping sites collected every ten days between April and September in the year 2014 comprise 240,572 female mosquito specimens (14 taxa in 8 genera), representing 24.14% of the 58 currently known mosquito species of Romania [19–25]. The screening results were used to identify potential mosquito vectors of *Dirofilaria* spp. in Romania and to better understand the temporal risk of transmission. Furthermore, after the detection of positive mosquito pools for 2014, blood samples of randomly selected dogs collected in each of the four mosquito sampling sites in 2016 were screened for *Dirofilaria* spp. DNA to evaluate the local prevalence of both filarioids in the definitive host.

## Methods

Details regarding the sampling methods, sampling sites and morphological mosquito identification were previously described [19]. The Danube Delta Biosphere Reserve Authority issued the research permits and corresponding approvals (9/25.04.2014, 10692/ARBDD/25.04.2014; 7717/ARBDD/28.04.2016, 11/28.04.2016). Mosquitoes were pooled per mosquito taxon, date and sampling site. This resulted in pools between 1 and 250 specimens at the maximum (mean = 113.58).

In addition, at the end of September/beginning of October in 2016, 36 dogs were sampled at the 4 mosquito sampling sites (2–15 dogs per site). These were very isolated and characterized by a few people with a small number of owned dogs. All locally available dogs allowing blood sampling with the assistance of the owner were sampled. The animals did not show any clinical symptoms. Blood samples from the cephalic vein of domestic dogs were collected in ethylenediaminetetraacetic acid at each of the four mosquito sampling sites and transported to the laboratory on dry ice. DNA of mosquito pools and single dog samples were extracted with a KingFisher™ Flex Magnetic Particle Processor using MagMAX™ Pathogen ribonucleic acid/DNA Kit (both Thermo Fisher Scientific, Waltham, MA, USA). The samples were screened with the previously described duplex real-time PCR assay to detect *D. repens* and *D. immitis* [7, 26]. This assay targets the mitochondrial cytochrome *c* oxidase subunit 1 and the nuclear *16S* rRNA gene fragment to differentiate both parasites. It is important to note that the number of sampled mosquitoes was very high, resulting in relatively huge pool sizes. This might result in reduced sensitivity of the real-time PCR, i.e. false negative results.

The program R [27] with four different packages was used for all analyses. *ggplot2* [28], *tidyr* [29] and *plyr* [30] were used to analyse and visualize the results. The functions of the package *binGroup* [31] were applied to calculate estimated infection rates (EIRs) and corresponding 95% confidence intervals (95% CI) per vector species and *Dirofilaria* species. Biased-corrected maximum likelihood estimation was used for point estimates and skewness-correction for 95% CIs. Significant differences were apparent from non-overlapping 95% CIs.

## Results

**Fig. 1:**
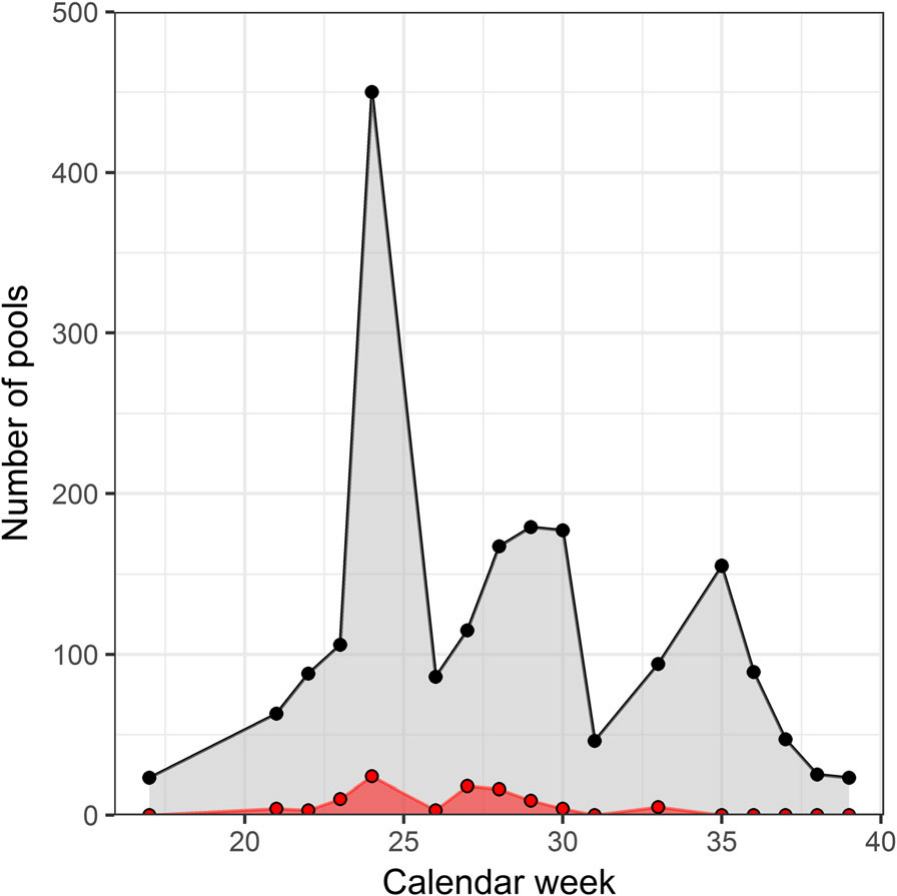
Pools tested per week (black) and the number of mosquito pools positive for *Dirofilaria* spp. (red) per calendar week in 2014 over all four sampling sites in the Danube Delta Biosphere Reserve (Romania)

Mosquito pools from 14 mosquito taxa in six different genera (*Uranotaenia*, *Culiseta*, *Culex*, *Coquillettidia*, *Anopheles* and *Aedes*) were screened for *Dirofilaria* spp. using a real-time duplex PCR (Table 1). From the 240,572 specimens, *Coquillettidia richiardii* (40.85%) and *Anopheles hyrcanus* (34.12%) were most frequent, followed by five species each between 3 and 8% of all mosquito specimens: *Culex pipiens* (*s.l.*)/*Culex torrentium*, *Aedes caspius*, *Culex modestus*, *Anopheles maculipennis* (*s.l.*) and *Ae. vexans*. Out of 2118 mosquito pools screened, 96 pools (4.53%) tested positive for *Dirofilaria* spp. DNA (EIR per 100 specimens = 0.041, 95% CI: 0.034–0.050), which further divided into 83 pools (3.92%) positive for *D. immitis* (EIR = 0.036, 95% CI: 0.029–0.044) and 23 pools (1.09%) positive for *D. repens* (EIR = 0.010, 95% CI: 0.006–0.014). In total, 10 pools (0.47%) were tested positive for both *Dirofilaria* species representing 10.42% of all pools tested positive for *Dirofilaria* spp. DNA. Thereby, the number of positive pools per calendar week was statistically significantly positively correlated with the number of screened pools per calendar week (*r*_S_ = 0.74, *P* < 0.001; Fig. 1).

**Table 1:** Mosquito taxa collected in the Danube Delta Biosphere Reserve (Romania) in 2014 with information on the number of screened mosquito specimens, tested pools, *Dirofilaria* spp. screening results and estimated infection rates (EIR) per 100 mosquito specimens with corresponding 95% confidence intervals (95% CI)

Mosquito species	No. of tested mosquito specimens	No. of tested mosquito pools	No. of mosquito pools positive for *Dirofilaria* spp. (% of tested pools)	No. of mosquito pools positive for *D. immitis* (% of tested pools)	EIR *Dirofilaria immitis* (95% CI)	No. of mosquito pools positive for *D. immitis* and *D. repens* (% of tested pools)	EIR *D. repens* (95% CI)
*Coquillettidia richiardii*	98,276	552	22 (3.99)	20 (3.62)	0.021 (0.013–0.032)	4 (0.72)	0.004 (0.001–0.010)
*Anopheles hyrcanus*	82,073	484	24 (4.96)	22 (4.55)	0.028 (0.018–0.041)	5 (1.03)	0.006 (0.002–0.014)
*Culex pipiens* (*s.l.*)/*Cx. torrentium*	18,421	217	9 (4.15)	9 (4.15)	0.051 (0.025–0.093)	0 (0.00)	0 (0.000–0.020)
*Aedes caspius*	13,729	197	9 (4.57)	7 (3.55)	0.053 (0.023–0.104)	3 (1.52)	0.022 (0.006–0.060)
*Culex modestus*	9534	166	0 (0.00)	0 (0.00)	0 (0.000–0.039)	0 (0.00)	0 (0.000–0.039)
*Anopheles maculipennis* (*s.l.*)	9380	207	20 (9.66)	17 (8.21)	0.206 (0.125–0.325)	6 (2.90)	0.066 (0.027–0.137)
*Aedes vexans*	7295	155	8 (5.16)	5 (3.23)	0.073 (0.027–0.164)	4 (2.58)	0.056 (0.018–0.134)
unidentified	1041	75	2 (2.67)	2 (2.67)	0.183 (0.036–0.576)	0 (0.00)	0 (0.000–0.315)
*Anopheles algeriensis*	697	34	1 (2.94)	1 (2.94)	0.126 (0.009–0.600)	0 (0.00)	0 (0.000–0.398)
*Aedes* spp.	71	12	1 (8.33)	0 (0.00)	0 (0.000–3.570)	1 (8.33)	1.263 (0.086–6.023)
*Aedes detritus*	31	5	0 (0.00)	0 (0.00)	0 (0.000–7.890)	0 (0.00)	0 (0.000–7.890)
*Culex* spp.	10	7	0 (0.00)	0 (0.00)	0 (0.000–25.441)	0 (0.00)	0 (0.000–25.441)
*Aedes flavescens*	5	2	0 (0.00)	0 (0.00)	0 (0.000–31.926)	0 (0.00)	0 (0.000–31.926)
*Aedes cinereus*	4	2	0 (0.00)	0 (0.00)	0 (0.000–39.179)	0 (0.00)	0 (0.000–39.179)
*Aedes hungaricus*	3	1	0 (0.00)	0 (0.00)	0 (0.000–40.888)	0 (0.00)	0 (0.000–40.888)
*Culiseta annulata*	1	1	0 (0.00)	0 (0.00)	0 (0.000–79.345)	0 (0.00)	0 (0.000–79.345)
*Uranotaenia unguiculata*	1	1	0 (0.00)	0 (0.00)	0 (0.000–79.345)	0 (0.00)	0 (0.000–79.345)
Sum resp. EIR (% of tested mosquito pools or 95% CI)	240,572	2118	96 (4.53)	83 (3.92)	0.036 (0.029–0.044)	23 (1.09)	0.010 (0.006–0.014)

*Dirofilaria immitis* was detected for eight and *D. repens* for six of the 14 screened mosquito taxa (Table 1). From all mosquito species collected with more than 50 specimens, only *Cx. modestus*, represented by more than 9000 specimens, was negative for *Dirofilaria* spp. DNA. The number of screened specimens per taxon was positively correlated with the number of analyzed specimens per taxon (*r*_S_ = 0.85, *P* < 0.001).

For the mosquito species with larger sample sizes (> 5000 specimens), allowing the calculation of reliable infection rates [32], *An. maculipennis* (*s.l.*) had highest rates of infection for both *Dirofilaria* species (*D. immitis*: EIR = 0.206, 95% CI: 0.125–0.325; *D. repens*: EIR = 0.066, 95% CI: 0.027–0.137). Non-overlapping confidence intervals indicate statistically significantly lower EIRs for the two most frequent mosquito taxa *Cq. richiardii* (*D. immitis*: EIR = 0.021, 95% CI: 0.013–0.032; *D. repens*: EIR = 0.004, 95% CI: 0.001–0.010) and *An. hyrcanus* (*D. immitis*: EIR = 0.028, 95% CI: 0.018–0.041; *D. repens*: EIR = 0.006, 95% CI: 0.002–0.014).

Between 2.85 and 5.49% of the tested mosquito pools per trapping site were positive for *Dirofilaria* spp. DNA (Table 2). *Dirofilaria immitis* was more prevalent than *D. repens* for all four sites. These mosquito screening results are also reflected in the analysis of domestic canids. At each of the four sites, dogs were tested positive for *Dirofilaria* spp. DNA (30.56% of all 36 screened specimens). Thereby, the proportion of positive dogs per site ranged from 20.00 to 50.00%. Both filarioid species had similar prevalence (*D. immitis*: 7 dogs, 19.44%, *D. repens*: 8 dogs, 22.22%), with a high proportion of co-infections (4 dogs, 36.66% of *Dirofilaria* spp. positive specimens).

**Table 2:** *Dirofilaria* spp. screening results of the four sampling sites in the Danube Delta Biosphere Reserve (Romania) with information on the number of screened mosquitoes (2014) and dogs (2016)

Trapping site	No. of tested mosquito specimens	No. of tested mosquito pools	No. of mosquito pools positive for *Dirofilaria* spp. (% of tested pools)	No. of mosquito pools positive for *D. immitis* (% of tested pools)	No. of mosquito pools positive for *D. repens* (% of tested pools)	No. of mosquito pools positive for *D. immitis* and *D. repens* (% of tested pools)	No. of tested dogs	No. of dogs positive for *Dirofilaria* spp. (% of tested dogs)	No. of dogs positive for *D. immitis* (% of tested dogs)	No. of dogs positive for *D. repens* (% of tested dogs)	No. of dogs positive for *D. immitis* and *D. repens* (% of tested dogs)
Dunarea Veche	48,423	386	11 (2.85)	8 (2.07)	5 (1.30)	2 (0.52)	7	3 (42.86)	3 (42.86)	1 (14.29)	1 (14.29)
Lake Rosulet	121,314	948	52 (5.49)	50 (5.27)	8 (0.84)	6 (0.63)	2	1 (50.00)	1 (50.00)	1 (50.00)	1 (50.00)
Letea	34,103	383	15 (3.92)	11 (2.87)	6 (1.57)	2 (0.52)	12	4 (33.33)	2 (16.67)	3 (25.00)	1 (8.33)
Sulina	36,732	401	18 (4.49)	14 (3.49)	4 (1.00)	0 (0.00)	15	3 (20.00)	1 (6.67)	3 (20.00)	1 (6.67)
Sum (% of tested mosquito pools or dogs)	240,572	2118	96 (4.53)	83 (3.92)	23 (1.09)	10 (0.47)	36	11 (30.56)	7 (19.44)	8 (22.22)	4 (11.11)

## Discussion

The circulation of *Dirofilaria* spp. in Romania has been known for several years, but was primarily demonstrated through monitoring of domestic and wild carnivores [8–15]. In contrast, information regarding the potential mosquito vectors was scarce [18]. Due to transportation problems, the lack of knowledge in particular applies to the DDBR [19]. Huge areas of the biosphere reserve are only reachable by boat. Results of the presented study confirm the local circulation of *D. immitis* and *D. repens* for all four sampling sites. *Dirofilaria* spp. DNA was detected in eight of the 14 analyzed mosquito species, indicating that 13.79% of the 58 currently known mosquito species of the country [19–25] have to be considered as potential vectors. This observation supports the assumption that a broad spectrum of mosquito taxa is potentially involved in the transmission cycles of *Dirofilaria* spp. in Europe with more than 20 species found infected so far [7, 18, 26, 33–48].

All mosquito species positive for *Dirofilaria* spp. DNA in the DDBR were classified as suspected vectors in at least one European xenomonitoring study [7, 26, 33–48], including *Ae. vexans* determined as potential vector in Romania before [18]. The number of positive pools per mosquito taxon was statistically positively correlated with the number of screened pools. Except for *Cx. modestus*, at least one pool of all mosquito species collected with more than 50 specimens was detected positive for one or both *Dirofilaria* species. More than 9000 *Cx. modestus* specimens were tested, but none of the pools were positive. However, the species was only identified as a potential vector in two European countries, Hungary [33, 38] and Moldova [26], which might allow the conclusion that the species is not the most important vector for *Dirofilaria* spp. in Europe.

In contrast, the analysis of a wide range of mosquito species again underlines the relevance of *An. maculipennis* (*s.l.*) in the local transmission cycles of dirofilariosis. Although trapped ten times less compared to the two most abundant species (*Cq. richiardii*, *An. hyrcanus*), this species had statistically significant higher infection rates. A similar observation was reported for Moldova [26]. In addition, positive pools of *An. maculipennis* (*s.l.*) were detected in Hungary [33], Italy [37], Austria [44], Germany [42, 46], Portugal [34] and Moldova [26], further underlying the relevance of *An. maculipennis* (*s.l.*) as vector of *Dirofilaria* species in Europe. This can partly be explained by the host-feeding patterns of the species, considered as a predominantly mammalophilic behavior [49, 50]. However, this also applies to other screened mosquito taxa (e.g. *Cq. richiardii*). Nevertheless, vector competence for *Dirofilaria* spp. is species-specific and can be affected by different factors. This includes the encapsulation/melanization of the parasite [51] or the increase of mosquito mortality linked to invasion of cells belonging to the Malphigian tubule [47, 52, 53]. Therefore, further vector competence studies are required to experimentally evaluate, which mosquito species are susceptible for *Dirofilaia* spp. infections [52, 54, 55].

The prevalence of *Dirofilaria* spp. in mosquitoes was positively correlated with the phenology of mosquitoes. A similar pattern was observed in Italy [47, 56] and Moldova [26], while no variation in the *Dirofilaria* spp. infection throughout the year in Portugal [34] and another study in Italy [35] has been observed. As previously discussed [26, 47, 57], the local risk for the transmission of *Dirofilaria* spp. is probably primarily driven by the abundance of the vector species and the presence of infected dogs.

Although the overall number of analyzed dog specimens was relative low (*n* = 36), infection rates between 20–50% indicate a high prevalence of *Dirofilaria* spp. in the four studied sites in the DDBR. Similar high values were found in several other studies in Romania [8–13]. Furthermore, there was a remarkably high proportion of co-infected dogs with more than 30% of all *Dirofilaria* spp. positive dogs infected with both helminths, *D. immitis* and *D. repens*. The finding is in agreement with a previous study from Romania, in which one quarter of all tested dogs were found to be infected with both *Dirofilaria* species [12]. This was also reflected in the screening results of mosquitoes from the same sampling sites. Although it cannot be ruled out that some of the detected co-infections in the mosquito pools are the result of different mosquito specimens infected with one or the other *Dirofilaria* species, the simultaneous detection of both parasite species in a high proportion of *Dirofilaria* spp. positive pools (10.42%) indicate a high probability of co-infected specimens. However, further studies are required to understand the risk of microfilaria transmission through these mosquitoes. The differences in the ecology of both *Dirofilaria* species within the mosquitoes as intermediate hosts are quite unknown. This includes potential competition, host specificity or general requirements for successful development [26].

## Conclusions

Recent studies on the circulation of *Dirofilaria* spp. in different eastern European countries highlight significant local circulation with a wide range of mosquito species involved as vectors (e.g. Belarus [7] and Moldova [26]). These findings are confirmed in the here presented results for the DDBR (Romania), identifying a high prevalence of *Dirofilaria* spp. in domestic dogs and several potential mosquito vector species. Further systematic monitoring studies including different components of the *Dirofilaria* spp. transmission cycle (mosquito vectors, dogs as definitive and humans as secondary hosts) should be implemented in eastern European countries to evaluate the local risk of human and canine dirofilariosis, allowing the implementation of effective surveillance and control measures.

## Abbreviations

DDBR: Danube Delta Biosphere Reserve
DNA: deoxyribonucleic acid
EIR: estimated infection rates
PCR: polymerase chain reaction
RNA: ribonucleic acid

## References

[1] Simon F, Siles-Lucas M, Morchon R, Gonzalez-Miguel J, Mellado I, Carreton E (2012). Human and animal dirofilariasis: the emergence of a zoonotic mosaic. Clin Microbiol Rev.

[2] Otranto D, Dantas-Torres F, Brianti E, Traversa D, Petrić D, Genchi C (2013). Vector-borne helminths of dogs and humans in Europe. Parasit Vectors.

[3] Pampiglione S, Rivasi F (2000). Human dirofilariasis due to *Dirofilaria* (*Nochtiella*) *repens*: an update of world literature from 1995 to 2000. Parassitologia.

[4] Poppert S, Hodapp M, Krueger A, Hegasy G, Niesen WD, Kern WV (2009). *Dirofilaria repens* infection and concomitant meningoencephalitis. Emerg Infect Dis.

[5] Salamatin RV, Pavlikovska TM, Sagach OS, Nikolayenko SM, Kornyushin VV, Kharchenko VO (2013). Human dirofilariasis due to *Dirofilaria repens* in Ukraine, an emergent zoonosis: epidemiological report of 1465 cases. Acta Parasitol.

[6] Harizanov RN, Jordanova DP, Bikov IS (2014). Some aspects of the epidemiology, clinical manifestations, and diagnosis of human dirofilariasis caused by *Dirofilaria repens*. Parasitol Res.

[7] Șuleșco T, Volkova T, Yashkova S, Tomazatos A, von Thien H, Lühken R (2016). Detection of *Dirofilaria repens* and *Dirofilaria immitis* DNA in mosquitoes from Belarus. Parasitol Res.

[8] Coman S, Bacescu B, Coman T, Parvu G, Dinu C, Petrut T (2007). Epidemiological and paraclinical aspects of canine dirofiloariosis. Lucr Stiinłifice Med Vet.

[9] Ciocan R, Dărăbuş G, Igna V (2010). Morphometric study of microfilariae of *Dirofilaria* spp. on dogs. Bull UASVM Vet Med.

[10] Ciocan R, Mederle N, Jacsó O, Tánczos B, Fok É (2013). Autochthonous cases of *Dirofilaria* in dogs from Timiș county (western part) Romania. Glob J Med Res.

[11] Mircean V, Dumitrache MO, Gyorke A, Pantchev N, Jodies R, Mihalca AD (2012). Seroprevalence and geographic distribution of *Dirofilaria immitis* and tick-borne infections (*Anaplasma phagocytophilum*, *Borrelia burgdorferi sensu lato*, and *Ehrlichia canis*) in dogs from Romania. Vector Borne Zoonotic Dis.

[12] Ionica AM, Matei IA, Mircean V, Dumitrache MO, D’Amico G, Gyorke A (2015). Current surveys on the prevalence and distribution of *Dirofilaria* spp. and *Acanthocheilonema reconditum* infections in dogs in Romania. Parasitol Res.

[13] Ciucă L, Musella V, Miron LD, Maurelli MP, Cringoli G, Bosco A (2016). Geographic distribution of canine heartworm (*Dirofilaria immitis*) infection in stray dogs of eastern Romania. Geospatial Health.

[14] Ionica AM, Matei IA, D’Amico G, Daskalaki AA, Jurankova J, Ionescu DT (2016). Role of golden jackals (*Canis aureus*) as natural reservoirs of *Dirofilaria* spp. in Romania. Parasit Vectors.

[15] Ionică AM, Matei IA, D’Amico G, Ababii J, Daskalaki AA, Sándor AD (2017). Filarioid infections in wild carnivores: a multispecies survey in Romania. Parasit Vectors.

[16] Manescu R, Barascu D, Mocanu C, Pirvanescu H, Mindria I, Balasoiu M (2009). Subconjunctival nodule with *Dirofilaria repens*. Chir Buchar Rom 1990.

[17] Popescu I, Tudose I, Racz P, Muntau B, Giurcaneanu C, Poppert S (2012). Human *Dirofilaria repens* infection in Romania: a case report. Case Rep Infect Dis.

[18] Ionică AM, Zittra C, Wimmer V, Leitner N, Votýpka J, Modrý D (2017). Mosquitoes in the Danube Delta: searching for vectors of filarioid helminths and avian malaria. Parasit Vectors.

[19] Török E, Tomazatos A, Cadar D, Horváth C, Keresztes L, Jansen S (2016). Pilot longitudinal mosquito surveillance study in the Danube Delta Biosphere Reserve and the first reports of *Anopheles algeriensis* Theobald, 1903 and *Aedes hungaricus* Mihályi, 1955 for Romania. Parasit Vectors.

[20] Prioteasa F-L, Falcuta E (2010). An annotated checklist of the mosquitoes (Diptera: Culicidae) of the Danube Delta Biosphere Reserve. Eur Mosq Bull.

[21] Prioteasa LF, Dinu S, Falcuta E, Ceianu CS (2015). Established population of the invasive mosquito species *Aedes albopictus* in Romania, 2012–14. J Am Mosq Control Assoc.

[22] Nicolescu G, Vladimirescu A, Ciolpan O (2002). The distribution of mosquitoes in Romania (Diptera: Culicidae). Part I: *Anopheles*, *Aedes* and *Culex*. Eur Mosq Bull.

[23] Nicolescu G, Vladimirescu A, Ciolpan O (2003). The distribution of mosquitoes in Romania (Diptera: Culicidae). Part II: *Culiseta*, *Coquillettidia*, *Ochlerotatus*, *Orthopodomyia* and *Uranotaenia*. Eur Mosq Bull.

[24] Nicolescu G, Vladimirescu A, Ciolpan O (2003). The distribution of mosquitoes in Romania (Diptera: Culicidae). Part III: Detailed maps for *Anopheles*, *Aedes* and *Culex*. Eur Mosq Bull.

[25] Nicolescu G, Vladimirescu A, Ciolpan O (2003). The distribution of mosquitoes in Romania (Dipera: Culicidae). Part IV: Detailed maps for *Coquillettidia*, *Culiseta*, *Ochlerotatus*, *Orthopodomyia* and *Uranotaenia*. Eur Mosq Bull.

[26] Șuleșco T, von Thien H, Toderaș L, Toderaș I, Lühken R, Tannich E (2016). Circulation of *Dirofilaria repens* and *Dirofilaria immitis* in Moldova. Parasit Vectors.

[27] R Core Team (2016). R: a language and environment for statistical computing.

[28] Wickham H (2009). ggplot2: elegant graphics for data analysis.

[29] Wickham H, Henry L (2017). tidyr: easily tidy data with “spread()” and “gather()” functions.

[30] Wickham H (2011). The split-apply-combine strategy for data analysis. J Stat Softw.

[31] Zhang B, Bilder C, Biggerstaff B, Schaarschmidt F (2012). binGroup: Evaluation and experimental design for binomial group testing.

[32] Walter SD, Hildreth SW, Beaty BJ (1980). Estimation of infection rates in populations of organisms using pools of variable size. Am J Epidemiol.

[33] Kemenesi G, Kurucz K, Kepner A, Dallos B, Oldal M, Herczeg R (2015). Circulation of *Dirofilaria repens*, *Setaria tundra*, and Onchocercidae species in Hungary during the period 2011–2013. Vet Parasitol.

[34] Ferreira CAC, de Pinho Mixao V, Novo MTLM, Calado MMP, Goncalves LAP, Belo SMD (2015). First molecular identification of mosquito vectors of *Dirofilaria immitis* in continental Portugal. Parasit Vectors.

[35] Latrofa MS, Montarsi F, Ciocchetta S, Annoscia G, Dantas-Torres F, Ravagnan S (2012). Molecular xenomonitoring of *Dirofilaria immitis* and *Dirofilaria repens* in mosquitoes from north-eastern Italy by real-time PCR coupled with melting curve analysis. Parasit Vectors.

[36] Bocková E, Iglódyová A, Kočišová A (2015). Potential mosquito (Diptera:Culicidae) vector of *Dirofilaria repens* and *Dirofilaria immitis* in urban areas of eastern Slovakia. Parasitol Res.

[37] Cancrini G, Magi M, Gabrielli S, Arispici M, Tolari F, Dell’Omodarme M (2006). Natural vectors of dirofilariasis in rural and urban areas of the Tuscan region, central Italy. J Med Entomol.

[38] Zittra C, Kocziha Z, Pinnyei S, Harl J, Kieser K, Laciny A (2015). Screening blood-fed mosquitoes for the diagnosis of filarioid helminths and avian malaria. Parasit Vectors.

[39] Bocková E, Rudolf I, Kočišová A, Betášová L, Venclíková K, Mendel J (2013). *Dirofilaria repens* microfilariae in *Aedes vexans* mosquitoes in Slovakia. Parasitol Res.

[40] Bravo-Barriga D, Parreira R, Almeida APG, Calado M, Blanco-Ciudad J, Serrano-Aguilera FJ (2016). *Culex pipiens* as a potential vector for transmission of *Dirofilaria immitis* and other unclassified Filarioidea in Southwest Spain. Vet Parasitol.

[41] Kurucz K, Kepner A, Krtinic B, Zana B, Földes F, Bányai K (2016). First molecular identification of *Dirofilaria* spp. (Onchocercidae) in mosquitoes from Serbia. Parasitol Res.

[42] Kronefeld M, Kampen H, Sassnau R, Werner D (2014). Molecular detection of *Dirofilaria immitis*, *Dirofilaria repens* and *Setaria tundra* in mosquitoes from Germany. Parasit Vectors.

[43] Rudolf I, Šebesta O, Mendel J, Betášová L, Bocková E, Jedličková P (2014). Zoonotic *Dirofilaria repens* (Nematoda: Filarioidea) in *Aedes vexans* mosquitoes, Czech Republic. Parasitol Res.

[44] Silbermayr K, Eigner B, Joachim A, Duscher GG, Seidel B, Allerberger F (2014). Autochthonous *Dirofilaria repens* in Austria. Parasit Vectors.

[45] Yildirim A, Inci A, Duzlu O, Biskin Z, Ica A, Sahin I (2011). *Aedes vexans* and *Culex pipiens* as the potential vectors of *Dirofilaria immitis* in central Turkey. Vet Parasitol.

[46] Czajka C, Becker N, Jöst H, Poppert S, Schmidt-Chanasit J, Krüger A (2014). Stable transmission of *Dirofilaria repens* nematodes, northern Germany. Emerg Infect Dis.

[47] Capelli G, Frangipane di Regalbono A, Simonato G, Cassini R, Cazzin S, Cancrini G (2013). Risk of canine and human exposure to *Dirofilaria immitis* infected mosquitoes in endemic areas of Italy. Parasit Vectors.

[48] Cancrini G, Scaramozzino P, Gabrielli S, Di Paolo M, Toma L, Romi R (2007). *Aedes albopictus* and *Culex pipiens* implicated as natural vectors of *Dirofilaria repens* in central Italy. J Med Entomol.

[49] Börstler J, Jöst H, Garms R, Krüger A, Tannich E, Becker N (2016). Host-feeding patterns of mosquito species in Germany. Parasit Vectors.

[50] Schönenberger AC, Wagner S, Tuten HC, Schaffner F, Torgerson P, Furrer S (2015). Host preferences in host-seeking and blood-fed mosquitoes in Switzerland. Med Vet Entomol.

[51] Zielke E (1993). Schutzmechanismen von Culiciden gegenüber Infestationen mit Filarien. Mitt Österr Ges Tropenmed Parasitol.

[52] Lai CH, Tung KC, Ooi HK, Wang JS (2000). Competence of *Aedes albopictus* and *Culex quinquefasciatus* as vector of *Dirofilaria immitis* after blood meal with different microfilarial density. Vet Parasitol.

[53] Serrao ML, Labarthe N, Lourenco-de-Oliveira R (2001). Vectorial competence of *Aedes aegypti* (Linnaeus 1762) Rio de Janeiro strain to *Dirofilaria immitis* (Leidy 1856). Mem Inst Oswaldo Cruz.

[54] Montarsi F, Ciocchetta S, Devine G, Ravagnan S, Mutinelli F, Frangipane di Regalbono A (2015). Development of *Dirofilaria immitis* within the mosquito *Aedes* (*Finlaya*) *koreicus*, a new invasive species for Europe. Parasit Vectors.

[55] Silaghi C, Beck R, Capelli G, Montarsi F, Mathis A (2017). Development of *Dirofilaria immitis* and *Dirofilaria repens* in *Aedes japonicus* and *Aedes geniculatus*. Parasit Vectors..

[56] Capelli G, Poglayen G, Bertotti F, Giupponi S, Martini M (1996). The host-parasite relationship in canine heartworm infection in a hyperendemic area of Italy. Vet Res Commun.

[57] Montoya-Alonso JA, Mellado I, Carreton E, Cabrera-Pedrero ED, Morchon R, Simon F (2010). Canine dirofilariosis caused by *Dirofilaria immitis* is a risk factor for the human population on the island of Gran Canaria, Canary Islands, Spain. Parasitol Res.

